# Endoscopic ultrasound-guided cyanoacrylate injection into the perforating vein for high-risk gastric varices

**DOI:** 10.1055/a-2333-9436

**Published:** 2024-06-12

**Authors:** Ahmed Youssef Altonbary, Fady Sabry, Hazem Hakim, Seham Seif

**Affiliations:** 1 68779 Department of Gastroenterology and Hepatology, Mansoura University, Mansoura, Egypt

Direct endoscopic injection of gastric varices using cyanoacrylate (CYA) is associated with significant adverse events such as bleeding from the post-injection ulcer, needle sticking in the varix, adherence of the glue to the endoscope, and embolization into pulmonary or systemic vessels 1 . Targeting the perforating vessel by endoscopic ultrasound (EUS) rather than the varix lumen may theoretically minimize the amount of CYA and thereby reduce complications 2 . A randomized controlled trial performed by our group concluded that EUS-guided CYA injection into the perforating veins achieved excellent technical success with a lesser amount of CYA, fewer number of sessions to obliteration, and fewer complications compared to direct endoscopic injection 3 .

A 62-year-old man with liver cirrhosis and splenomegaly was referred to our facility for management of large gastric varices diagnosed during screening upper endoscopy. The patient had no previous attacks of hematemesis or melena. His laboratory studies were unremarkable apart from pancytopenia associated with hypersplenism. In a trans-esophageal approach, the perforator feeding veins were targeted via EUS-guided fine-needle aspiration with a 19G needle. The needle’s tip position inside the vessel was confirmed by injection of 1 ml saline followed by injection of a (1:1) mixture of 2-octyl-cyanoacrylate and lipiodol under real-time EUS guidance and then flushing by saline before the needle was withdrawn ( Fig. 1 ). The evolving clot inside the perforator feeding vessel was visualized under real-time EUS, and the immediate effect on Doppler flow inside the varix was assessed ( Video 1 ). After the procedure, the patient was observed for 2 hours in the recovery room before being discharged. An endoscopic examination repeated after 3 months confirmed eradication. No adverse events were reported during or after the procedure.

**Fig. 1:**
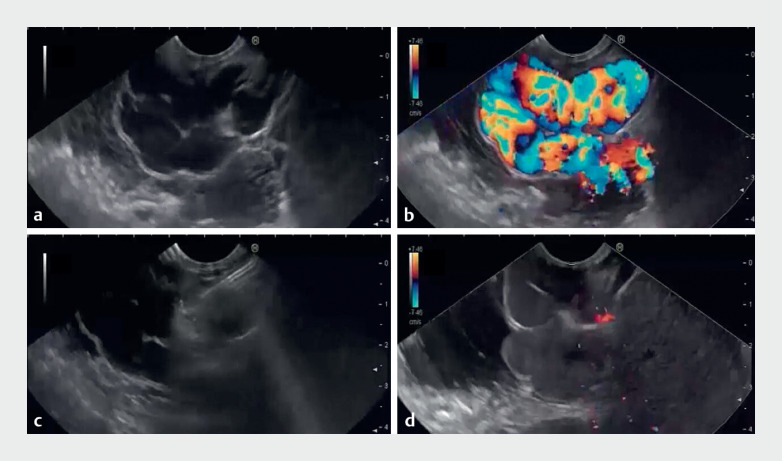
Endoscopic ultrasound-guided perforating vein injection of gastric varices. **a** A large gastric varix, 4.6 × 3.6 cm. **b** Color Doppler flow inside the varix. **c** Targeting feeding vessel with a 19G needle, and clot formation at feeding vessel after injection of (1:1) mixture of 2-octyl-cyanoacrylate and lipiodol. **d** No flow inside the gastric varix immediately after injection.

Endoscopic ultrasound-guided perforating vein injection of gastric varices.Video 1

In conclusion, given the high cost of vascular coils, EUS-guided CYA injection into the perforating veins could be a cost-effective and safe alternative in the treatment of high-risk gastric varices.

Endoscopy_UCTN_Code_TTT_1AO_2AD

## Notes

Endoscopy E-Videos https://eref.thieme.de/e-videos
*E-Videos* is an open access online section of the journal *Endoscopy* , reporting on interesting cases and new techniques in gastroenterological endoscopy. All papers include a high-quality video and are published with a Creative Commons CC-BY license. Endoscopy E-Videos qualify for HINARI discounts and waivers and eligibility is automatically checked during the submission process. We grant 100% waivers to articles whose corresponding authors are based in Group A countries and 50% waivers to those who are based in Group B countries as classified by Research4Life (see: https://www.research4life.org/access/eligibility/ ). This section has its own submission website at https://mc.manuscriptcentral.com/e-videos .
